# Families’ Stressors and Needs at Time of Cardio-Pulmonary Resuscitation: A Jordanian Perspective

**DOI:** 10.5539/gjhs.v6n2p72

**Published:** 2013-12-01

**Authors:** Rami Masa’Deh, Ahmad Saifan, Stephen Timmons, Stuart Nairn

**Affiliations:** 1 School of Nursing, Applied Science Private University, Amman, Jordan; 2 School of Health Science, University of Nottingham, UK; 3 Queen’s Medical Centre, School of Nursing, Midwifery & Physiotherapy, University of Nottingham, UK

**Keywords:** relative, resuscitation, need, witness, stress

## Abstract

**Background:**

During cardio-pulmonary resuscitation, family members, in some hospitals, are usually pushed to stay out of the resuscitation room. However, growing literature implies that family presence during resuscitation could be beneficial. Previous literature shows controversial belief whether or not a family member should be present during resuscitation of their relative. Some worldwide association such as the American Heart Association supports family-witnessed resuscitation and urge hospitals to develop policies to ease this process. The opinions on family-witnessed resuscitation vary widely among various cultures, and some hospitals are not applying such polices yet. This study explores family members’ needs during resuscitation in adult critical care settings.

**Methods:**

This is a part of larger study. The study was conducted in six hospitals in two major Jordanian cities. A purposive sample of seven family members, who had experience of having a resuscitated relative, was recruited over a period of six months. Semi-structured interview was utilised as the main data collection method in the study.

**Findings:**

The study findings revealed three main categories: families’ need for reassurance; families’ need for proximity; and families’ need for support. The need for information about patient’s condition was the most important need. Updating family members about patient’s condition would reduce their tension and improve their acceptance for the end result of resuscitation. All interviewed family members wanted the option to stay beside their loved one at end stage of their life. Distinctively, most of family members want this option for some religious and cultural reasons such as praying and supplicating to support their loved one.

**Conclusions:**

This study emphasizes the importance of considering the cultural and religious dimensions in any family-witnessed resuscitation programs. The study recommends that family members of resuscitated patients should be treated properly by professional communication and involving them in the treatment process. The implications concentrate on producing specific guidelines for allowing family-witnessed resuscitation in the Jordanian context. Finally, attaining these needs will in turn decrease stress of those witnessing resuscitation of their relative.

## 1. Introduction

There is a growing expectation that family members should have the option to stay at the bedside of their loved one during Cardio-Pulmonary Resuscitation (CPR). Witnessing resuscitation was initially explored by the Foote Hospital in Michigan, USA (Hanson & Strawser, 1992). Foote Hospital carried out a study examining family-witnessed resuscitation (FWR) over nine years. On more than one occasion, patients’ relatives asked to be present during the CPR process. As a result of relatives’ requests to witness CPR, healthcare providers asked the hospital’s policies department to create a new policy to prevent the attendance of patients’ relatives. Consequently, a survey to assess family wishes and requests for witnessing CPR was developed. More than 70% of people surveyed reported that they wished that they had been present during the resuscitation (Hanson & Strawser, 1992). Three years later, a new policy for FWR was implemented in the Foote Hospital (Hanson & Strawser, 1992). The policy stated that family should be asked to decide whether they wanted to be present during CPR or not. If relatives wanted to witness the patient’s CPR, support staff accompanied them before, during, and after CPR. A subsequent evaluation was conducted. Of 47 respondents who had been present during CPR, 76% of them said that their adjustment to the death was made easier by witnessing the CPR of their relative. A further 64% believed that their presence had been helpful to the patient (Doyle et al., 1987). The increase in requests from relatives to witness resuscitation, in addition to the demand from health professionals to have clear guidelines for organising this issue, led to resuscitation councils around the world producing guidelines. For example, the Resuscitation Council in the UK and The American Heart Association have developed guidelines for the purpose of advocating patients’ families’ right to be present during CPR (American Heart Association, 2006). Additionally, FWR has been supported by nursing and medical organisations such as the Royal College of Nursing and the British Association for Accident and Emergency Medicine (Axelsson et al., 2010; Madden & Condon, 2007). Recently, the European Federation of Critical Care Nursing Association, the European Society of Paediatrics and Neonatal Intensive Care and the European Society of Cardiology Council on Cardiovascular Nursing and Allied Professions jointly formulated a position statement on the presence of family members during CPR (Fulbrook, Albarran, & Latour, 2005). However, some countries have not yet implemented FWR polices.

After FWR was adopted in many hospitals around the world, there was a need to focus not only on the patient’s needs but also on the family members’ needs during FWR. Studies investigating this issue reported that critically ill patients’ relatives needed information, reassurance, support, comfort and being near their loved ones (Hinkle & Fitzpatrick, 2011; Verhaeghe, Defloor, Zuuren, Duijnstee, & Grypdonck, 2005). After recognition of the families’ needs and changing the priorities of care delivery, family members started to be given more space and freedom to participate in caring for their loved ones. Paediatric and maternity departments were among the first to embrace family-centred care (Baskett & Lim, 2004; Gamell et al., 2011). Baskett and Lim (2004) indicated that giving more freedom for patients and their relatives would require healthcare professionals to accommodate relatives’ presence during different situations. This might result in further demands for the presence of relatives during such procedures as resuscitation (Baskett & Lim, 2004). Exploring this issue further, Davidson, Jones and Bienvenu (2012) showed that having a family member in one of the critical care units produces a number of psychological and social stressors for both the patients and family members. Strong emotions, shock, denial, guilt and fear of the loss of the family member were the stressors reported by family members (Verhaeghe et al., 2005). Therefore, it is crucial to assess and meet the needs of family members of critically ill patients.

Assessing the needs of families’ of critically ill patients has been discussed by several authors (Al-Hassan & Hweidi, 2004; Davidson et al., 2012; Hupcey, 1999; Molter, 1979; Verhaeghe et al., 2005). Molter (1979) developed the Critical Care Family Needs Inventory (CCFNI), which consists of a list of 45 needs that family members could rate on 4-point Likert scales. These 45 needs were divided into five sub-scales: the need for support, comfort, information, proximity and assurance. Using CCFNI, Molter examined the needs of 40 family members of critically ill patients. Statistically, family members’ responses were analysed and the results showed that family members need to feel that there is hope; to feel that hospital personnel care about the patient; to know the prognosis; to receive information about the patient once per day; and family members need to see the patient frequently.

Molter’s study is imperative for the purpose of the present study. Firstly, Molter examined families’ needs in ICU, a critical care area. Secondly, this is the first study that considered asking the ICU nurses to treat the critically ill patient within the context of their families. Thirdly, similar findings were produced by several studies that adopted Molter’s CCFNI. However, there are some weaknesses in Molter’s study. The instrument of Molter’s study was originated from a survey of ICU nurses. This gives an idea that the critically ill patients’ families’ needs were determined by nurses, but not by families. Utilizing such structured data collection method might reduce the flexibility and the possibility of getting a wider perspective on the topic. Therefore, it is important to give more space to participants in order to express their feelings and state their unique experience when investigating this issue.

Holden, Harrison and Johnson (2002) reviewed several studies that examined critically ill patients’ families’ needs. Authors identified three basic needs, the need for information, the need for support from hospital staff and the need for hope. However, the researchers stated that most of the reviewed studies in this field adopted a quantitative approach and used CCFNI to collect their data (Holden et al., 2002). Additionally, it was also clear that most of the literature in this field have been conducted in Western countries and none in an Arab Muslim community, even though culture and religion background may affect the way that people cope with their life events. The excessive using of quantitative designs to examine FWR was thought to produce short answers and limited findings (Hanson, 2008; Strong, 1992). Many researchers suggested that there is a definite need for more qualitative research about FWR (Halm, 2005; Moreland & Manor, 2005; Redley, Botti, & Duke, 2004; Walker, 2008). It is important to adopt a qualitative approach in this study, as this is the first time this topic has been examined in Jordan. The use of qualitative methodology allows for discovery of themes from the data rather than verification of pre-existing themes (Ambert, Adler, Adler, & Detzner, 1995). This inductive approach means that the research can focus upon what the respondents find important, and not what pre-existing research dictates as important (Ambert et al., 1995). Adopting a qualitative approach provides a more holistic perspective about the subject under investigation (Strong, 1992).

In a study conducted in Jordan, Al-Hassan and Hweidi (2004) used Molter’s CCFNI to examine the needs of 158 family members who were visiting their critically ill relatives. The researchers reported that the majority of the respondents perceived 16 need statements as important or very important. The needs for assurance, information and proximity were ranked the highest. The need to receive information about the patient’s condition was the most important need for most of the respondents. Interestingly, needs for support and comfort were the lowest amongst the relatives’ needs. This was explained by the fact that Jordanian family members received greater support from other relatives (Al-Hassan & Hweidi, 2004). Al-Hassan and Hweidi’s study was of the few studies that evaluated the Jordanian critically ill patients’ families’ needs. There are several specific Jordanian cultural, social and religious issues were raised in this study. Most importantly, although this study used the CCFNI which was used by several Western studies, the authors reported some differences between Jordanian families’ needs and the needs of Western families. Despite the raising of many cultural and religious issues in this study, Al-Hassan and Hweidi failed to address families’ special cultural and religious needs. This might have resulted from their use of a quantitative design and using an instrument that was based on studies conducted to assess Western families’ needs.

Despite the presence of several studies that focused on FWR (Al-Mutair, Plummer, & Copnell, 2012; Grice, Picton, & Deakin, 2003; Mangurten et al., 2006; Meyers et al., 2000; Ong, Chan, Srither, & Lim, 2004), none of these studies considered families’ needs during this critical situation. Rather, the focus of most of the previous studies was on answering the question of “should family members have the option of witnessing CPR or not?” Moreover, all the reviewed studies were conducted in Western communities and no studies have been conducted to examine the effects of other cultural and religious values, particularly the Islamic religion and Arabic culture, on the issue of FWR. This study therefore highlights the influences of the Islamic religion and Arabic culture on family members’ perceptions and beliefs about FWR. The overall aim of this study is to identify family members’ needs during CPR. By identifying these needs and evaluating how they are being met, total patient care will involve the family and will in turn decrease their stress. To achieve this aim, this study tried to answer the following questions:

- What are the family members’ needs when having family members in the resuscitation room?
- What is the effect of cultural and religious issues on FWR of their loved one, within the Jordanian context?

## 2. Methods

The stress and coping model identified by Lazarus and Folkman (1984) suggests that stress may result from an imbalance between individual stressors and their resources to buffer them. Accordingly, there is a need to investigate family members’ needs during CPR of their relative. Qualitative research is particularly effective when there is a need for more understanding of social phenomena like FWR (Bryman, 2012). By adopting a qualitative research method, the focus of this study is on understanding the families’ needs when having a loved one facing death. Face-to-face semi-structured interviews were selected as a data collection method. This method allows the gathering of a greater amount of in-depth information thus enable the researcher to follow-up and probe incomplete, vague, or ambiguous responses (Maxwell, 2012). An interview guide was developed derived from the literature review and the authors’ experience in this field. This guide is presented in following box. The time spent in each interview ranged from 40 to 60 minutes.

### 2.1 Setting

Jordan is a small country situated in the Middle East, with a population of around 5.6 million. Health conditions in Jordan are among the best in the Middle East (USAID in Jordan, 2006). The healthcare system in Jordan consists of four sectors: private, university, military and public (Al-Hassan & Hweidi, 2004). Death from cardiovascular conditions forms 42% of deaths in Jordan. According to Khoury, Massad and Fardous (1999), the majority of cardiovascular deaths were recorded as cardiac and respiratory arrest, which represents the mode and not the cause of death. This reflects the importance of having an effective system to deal with this type of patient. Active CPR is a basic part of this system.

The study took place in six hospitals (two public, two private, and two university hospitals) situated in the capital of Jordan (Amman) and Irbid. These hospitals were chosen for several reasons. Firstly, 65% of the Jordanian population live in Amman and Irbid. Secondly, Amman is the central city in Jordan; Jordanians come from all over Jordan to Amman seeking health services.

**Interview schedule for interviewing family members**

- To get us started, I wonder if you would mind telling me a bit about yourself?
- Could you please tell me about your experience in detail?
- What is the relationship between you and your relative?
- Could you tell me about the attitudes of health professionals toward you and your family before, during and after your experience?
- Do you believe that family members should be able to be with their loved one just before death?
- Is it important to you that relatives were present during CPR? Why? Why not?
- Do you think family presence during CPR helped the treatment process? Did it help family members? How?
- In the literature, there are many concerns regarding family presence during CPR that have been raised by healthcare providers. Could you tell me your comments on these claims?
- What are families’ responsibilities during resuscitation? And what could the family have done or said to make the experience better for the patient?
- Do you believe that cultural and religious issues affect family presence during CPR of their loved one? How so?
- What is the effect of religion and culture on the families’ need to stay in the resuscitation room during CPR?
- How do the religion and culture affect the health professionals’ perceptions about the presence of family during CPR?
- How does the Arabic culture affect acceptance or refusal of family presence during CPR?
- What are the spiritual needs of the Muslim patients, in this context?
- What are the spiritual needs of Muslim patients’ families?
- Do you have any suggestions to better meet the needs of family members when they have a relative in the resuscitation room?

By conducting this study in critical care settings, the aim is to shed light on the specific features of these settings and their effects on families’ needs during CPR. Patients in such settings are at high risk for CPR, which makes the relationship between the critical care professionals, and patients and their relatives, different from relationships in other departments.

### 2.2 Gaining Access to the Clinical Setting

Each hospital had its own research ethics committee. A formal letter was presented with the ethical application for each hospital included in this study and to the Human Resources Centre in the Ministry of Health. Each application was accompanied by a full proposal. Ethical approval was gained from all the hospitals included.

### 2.3 Ethical Considerations

Participants have the right to decide voluntarily whether to participate in a study, without the risk of any penalty or prejudicial treatment (Polit & Beck, 2010). In this study, the expected participants had complete freedom to participate or to refuse participation in the research. The participants were given the choice to participate in this study, and to terminate their participation at any time.

The issue of privacy is invariably linked to the issue of anonymity and confidentiality in the research process (Bryman, 2012). In this study, patients’ relatives were met with in the clinical area, and a general explanation about the study and its main objectives was presented to them. An invitation letter was given to all of them, and they were asked to return it to nursing reception if they agreed to be interviewed. All participants were informed that their responses would be treated confidentially, and their account would always be anonymous. In this study, each participant was asked to fill in a consent form before each interview. This form was accompanied by an information sheet explaining the objectives and the main goal of the study. Moreover, the interviewees were told that the interview would be recorded and their quotes might be used in this study. A consent form was then given to the interviewee to sign. All interviewees accepted using the digital recorder.

### 2.4 Population and Sample

The study involved family members who had experienced one of their relatives undergoing CPR in any of the selected critical care settings. Due the fact that the end result of CPR may influence the reaction of family members (i.e. when CPR ends in death, family members become more stressful and traumatised when compared to a successful CPR), interviewing family members with both kinds of experiences was chosen in order to produce more in-depth results.

According to Mason (2002), purposive sampling means selecting groups or categories to study on the basis of their relevance to the research questions, the theoretical position and analytical framework. Consequently, it was thought that the purposive sampling could provide a good understanding of this phenomenon.

Patients’ relatives were chosen according to the following inclusion criteria:

- Being adult.
- Relative of patients in critical care settings.
- Relative of patients who have been exposed to CPR effort, either CPR succeed of failed.

At the end, seven family members were interviewed. Their demographic characteristics are shown in the next table (Table 1).

**Table 1:** The demographic characteristics of the interviewed family members (participants)

No.	Pseudonyms of the family members	Sex of the family members	Age of the family members	Education level of family members	Relation of the family members to the patient	CPR result	Did the family member witness the CPR?	Period after CPR
1.	Abu Yassin	M	28	Bachelors	Son	Survival	Yes	3 months
2.	Abu Rashid	M	41	Secondary	Brother	Survival	Yes	3 months
3.	Abu Isa	M	26	Diploma	Son	Survival	No	4 months
4.	Abu Hammad	M	42	PhD	Son	Death	No	6 months
5.	Om Adnan	F	50	Primary	Daughter	Death	No	3 months
6.	Abu Thaer	M	45	Secondary	Son	Death	No	9 months
7.	Abu Ali	M	35	Bachelors	Brother	Death	No	4 months

### 2.5 Recruitment Strategy

The researcher asked the head of department of each critical care unit about patients who had been exposed to CPR attempts before; and for the very critically ill patients who were expected to be resuscitated soon. This was done without getting any specific information about the patients’ conditions or names. One of the researchers went with a head of department or a senior staff member who worked in each unit to speak with patients’ family members. A brief explanation about the study and its importance was given to patients’ relatives. Permission for future contact was given by many of these relatives. The researcher kept in contact with the heads of critical care units about the conditions of these patients. Family members of patients that have had CPR were contacted two to three months after CPR and an invitation letter was posted to each of them.

In the existing literature, only three studies examined the attitudes of family members after real experience of CPR of one of their loved ones qualitatively (Wanger, 2004; Weissman, 2004; Woning, 1999). In two of these studies Woning (1999) and Wanger (2004), only five or six family members were interviewed. Seven family members were interviewed in this study. The sensitivity of the subject, particularly to Arabic Muslim people, reduced the chances of finding more than this number. Many invitation letters were distributed to family members in critical care settings. Although most of them stated that they were happy to be contacted in the future, only a few of them sent their contact details back to the researcher. Additionally, some family members who gave the researcher their contact details were not happy to talk about their experience. Despite the small sample size in the current study, the interviews were long and therefore generated new, relevant, useful data and nonetheless provided useful counterpoints and an in-depth analysis of patients’ relatives’ responses. The main aim of this qualitative study is to produce in-depth information about the phenomena being studied rather than generalisable information. Arabic pseudonyms were used instead of the participants’ names in order to maintain confidentiality. All of the interviewed family members were Muslims and were broadly middle class.

### 2.6 Data Analysis

Once the transcription was finished, the data were transferred to the computer by using the NVivo software which is one of the most popular software packages used by health researchers (Kopelman, 2006). The data was analysed by using the thematic analysis technique as identified by Braun and Clarke (2006) who stated six phases for thematic analysis. These steps are: familiarization with the data, generating initial codes, searching for themes, reviewing themes, defining and naming themes and finally producing the report.

## 3. Findings

The next three sections stats the themes resulting from this study. Quotes from participants are presented under each theme as examples.

### 3.1 Needs for Reassurance

In previous studies, the need for reassurance centred on relatives’ need to get accurate information about the condition of their loved one. The need for information and knowledge seems to be greatest (Bailey, Sabbagh, Loiselle, Boileau, & McVey, 2010; Verhaeghe, et al., 2005). Patients’ relatives need to get specific information about the condition, prognosis, and precise treatment of the patient (Bailey, et al., 2010). Davis (1994) stated that providing relatives with such information is important and should be presented in an understandable manner. Due to the fact that the result of CPR is often unpredictable, it is not easy for healthcare providers to communicate with relatives of a patient having CPR.

In this study, screens were the basic source of information about what was happening in the resuscitation room. This may help relatives to understand what is happening in CPR. It was clear that all of the relatives believed in the importance of keeping them up to date about the condition of their loved one. The families’ limited knowledge about the condition of their relative during CPR may lead them to underestimate the seriousness of the incident. This was thought to increase the difficulty of telling families about CPR outcomes. Lack of knowledge was also thought to produce a state of exaggerated hope by family members. Death after CPR is still a very common outcome. Even after survival, the patient’s condition may still be at risk of complications such as brain damage or recurrent cardiac arrest. For example one relative said:

…healthcare providers have to be more honest with family members. They have to put the family in the real situation of their patient to take appropriate actions. They should not provide an unreal picture and hide what has happened. This might lead the family to think that this is a simple condition, and then they discover that the condition is much worse than their imagination… (Abu Isa)

Family members interviewed implied that there was no direct conversation with the CPR team during CPR. Rather, communication mainly took place after the end of the procedure. This pushed patients’ relatives to find alternative methods to find out about their loved one’s condition. Nearly all the relatives interviewed talked about several ways of trying to observe the resuscitation efforts. Even family members who had witnessed CPR did not attend the initial stage of CPR. They stayed outside the CPR room for at least the first five to fifteen minutes. Then, they started to be curious about what had happened to their relative. Many relatives, for example, stood in front of the door of the resuscitation room to see what happened inside the room, especially when health professionals entered or left the room. Other relatives tried to overhear conversations between health professionals. Most relatives kept asking any staff leaving the room about the condition of their relative.

….When any of the nurses/doctors went in and out of the room, we asked them to reassure us. So, we were asking a lot. We asked more than 10 or 15 time about our father… (Abu Isa)

Several ways of looking for information were also identified. Four of the interviewed relatives thought that the presence of other relatives or friends with a health background was perceived as helpful. The presence of such a person was thought to improve families’ trust and was seen as a resource of information about the patient’s condition. This person, furthermore, assisted family members in controlling their feelings, and then taking right decisions as described by some of the interviewed relatives.

…The doctors gave general answers…But my friend has good health background and he told us that my father had an acute clot and needed an immediate cardiac catheterisation and he tried to re-assure us. He told us that the situation is critical but at the same time, he told us that this was not frightening; and this had happened to a lot of people… (Abu Isa)

Several relatives stated that they tried to interpret professionals’ verbalisations and facial expressions. Other relatives tried to analyse machine signals as a method of seeking the truth about the patient’s condition. For example:

…I was looking for their expressions and waiting for an answer from them… (Abu Hammad)

…There is like a monitor that gave signs of the working of the heart. I was optimistic when I saw these signs raised. At the beginning, it was a red line, but after giving her electric shocks, I felt that it started to move… (Abu Thaer)

From the above quotation, it is likely that the patient’s relative was not able to interpret the monitor. However, he was trying to find answers for questions that were in his mind. He might also have been seeking hope. The behaviour of information-seeking was reported in the literature by Weslien, Nilstun, Lundqvist and Fridlund (2006) who interviewed patients’ relatives and asked them about their perceptions regarding FWR. Some of the relatives listened to professionals’ exchanges and watched the cardiac monitor. By doing this, the relatives stated that they realised the seriousness of the patient’s condition (Marita Weslien et al., 2006).

Additionally, after interviewing a group of family members, Wanger (2004) reported one major theme ‘‘should we go or should we stay?’’. This theme consists of three phases. During the first phase, relatives recognised that something bad had happened with the patient. They stayed beside the patient until realising that health professionals had started to the most intensive phase of resuscitation. During the second phase, patients and their relatives experienced a crisis. They tried to enter the resuscitation room. In this phase, family members wanted to get information about the condition of their loved one, which led many relatives to find alternative information-seeking methods. In the last phase, family members started to break the rules and try to enter the room (Wanger, 2004). This seems similar to the attitudes of the patients’ relatives in the current study, as the relatives interviewed initially followed the health professionals’ directions and left the room. Family members then tried to get any information about their loved one’s condition. After a period of time, especially if nobody gave them sufficient information about the patient’s condition, they started to find other ways of getting information. This, sometimes, culminated in breaking the rules and entering the resuscitation room.

At time of CPR and sudden death, respect for patient autonomy and the intention to honour decisions to decline unwanted treatments should be conveyed to the family. Andreae (2010) explained that it is important to keep families informed, at the earliest possible opportunity, of any signs of deterioration in their loved one’s condition. To do this, Baskett et al. (2005) illustrated that it is a duty of the senior doctor or the team leader to communicate with family members about patients’ prognosis and the possible outcomes. Baskett et al. (2005) preferred that the team leader be accompanied by an experienced nurse who may be a great comfort for the patient and relatives. Farrell (1989) went further to suppose that the person who confirmed the death of the patient to the relatives should be a staff member with whom the relatives have had prior meaningful contact. This is expected to improve families’ acceptance of the outcomes of CPR. This may also create a trust relationship between family members and healthcare professionals.

In conclusion, the results of this study showed that keeping families updated about their patient’s condition helps in improving the relationships between health professionals and relatives and will bring their expectations to the reality. This may also improve trust and make families happier with the level of services presented to their loved one.

### 3.2 Needs for Proximity

Brysiewicz and Uys (2006) examined healthcare professionals’ ability and skills to deal with sudden death. They found that families appreciate being kept as close as possible to their loved one. The families’ need for proximity was raised in the literature. Walters (1995) and Jamerson et al. (1996) qualitatively examined the experience of family members of critically ill patients. The need for proximity was significant, meaning that family members physically want to stay with their loved one. This includes not only being in the unit or being around, but also the ability to talk to and touch the patient. Meeting the need for proximity seems to be a part of meeting families’ needs for reassurance and support. Giving family members the opportunity to hold hands and speak to the resuscitated person can ultimately help the grieving process (Walker, 2013).

In the current study, family members found it difficult to explain why they wanted to be present during CPR. They thought that needing to be with their loved one was part of human nature. Therefore, some relatives believed that excluding family members during CPR is against this nature. However, it should be considered that CPR is not just a place for caring about emotions. Abu Isa, for instance, stated that he just wanted this opportunity, but he did not know why.

…The person feels that he has to be there and do something, but not sitting at home or on the chair in the hospital. You have to be in the room even if you are sure that you cannot help… (Abu Isa)

In a similar view, one of the relatives said that he was used to being with his mother. He asked why he had to leave her at that difficult time. This seems to be in agreement with one of the fundamental principles of holism.

…I persisted in letting her feel that I was beside her, because she was used to finding me beside her. Really, I think that when she was listening to my voice and feeling me around her, this could improve her psychological status… (Abu Rashid)

Many studies found that staying beside the patient and being able to speak to and see them regularly are essential family members’ needs (Lee & Lau, 2003; McKiernan & McCarthy, 2010). Even in very intense incidents, family members need to be near the patient (Blom, Gustavsson, & Sundler, 2013). This might explain the relatives’ statements in this study, as they only wanted to be with their loved ones.

Among the relatives interviewed, there was an agreement that religion is an important issue when dealing with CPR. This is a unique finding for the present study. Religion was used by staff to manage relatives during CPR, and by families to control their feelings. Praying for God to assist the patient and to give the relative patience was among the families’ most important behaviours during CPR. It was mentioned that prayer could be done anywhere, and not necessarily beside the patient. Being close to the patient, however, was viewed as a way of enhancing prayer. Some relatives openly stated that prayer is better when done beside patient.

…Praying is not only beside a patient. But it is confirmed about the Prophet Muhammad that he was visiting his mother’s grave and cried (as she did not die a Muslim). Although his mother passed away 40 to 50 years before…but being close to the place, gives feeling of emotional proximity. Being close to the place encourages praying or encourages doing something… (Abu Yassin)

Several benefits were expected to be achieved through meeting families’ need for proximity. Being close to the loved one provides a feeling of trust. Presence in the resuscitation room shows family members that everything possible is done for the patient. In contrast, three relatives stated that not allowing FWR would mean that there is something to be hidden. However, it could be that health professionals may be not ready for this presence. The resuscitation room may need more preparation. It is important to find answers for relatives’ questions in this field.

…I think that this is my right. If they do not allow me to enter, I would have a negative view about hospital and I would feel that there is something wrong; there is a problem… (Abu Yassin)

### 3.3 Needs for Support

Brysiewicz and Uys (2006) show the importance of supporting family members in acute crisis. They stated that family members should not be kept alone in case of sudden death. Hanson and Strawser (1992) conducted a report about nine years of FWR experience in the Foote Hospital. The researchers recommended providing appropriate support for family members and stated that by providing such support, some stressors regarding FWR may be reduced (Hanson & Strawser, 1992).

The issue of support was also highlighted in the current study. CPR was seen as a very difficult incident. Mixed feelings were raised by all of the relatives when they described their emotions during CPR. Nearly all of the relatives did not hide their fears during their experiences. Patients’ relatives talked about the pressure they felt during CPR. Therefore, family members are in need of considerable support during CPR.

…You can’t really explain your feelings in such a situation. The whole scenario is really very scary… (Abu Yassin)

The results of this study showed that patients’ relatives did not receive enough support during CPR. This had several aspects. Firstly, it was explained that critical care units, especially in public and university hospitals, did not have dedicated waiting rooms for relatives. Secondly, the way of informing relatives about the end results of CPR, especially in the case of death, seemed inappropriate. It was stated that the leader of the CPR team usually went out to family members, and then told them about the end results. This happened either in an open department or in a corridor, as reported by the participants. The privacy of family members was not considered. This appeared to restrict families from expressing their feelings after CPR. The team leader, who had the responsibility to communicate with families, was usually a physician, who broke the bad news alone. However, the question arises of whether physicians are qualified to perform this job alone.

…I remembered that the doctor stayed with us for one to two minutes. After that, he told us you are a believer and you have to tolerate this; your mother passed away and you should tolerate this news. Then, we walked away and left us thrown into unknown… (Abu Rashid)

The above statements contradict the recommendations that family members should be supported during CPR (Marrone & Fogg, 2003; Walker, 2013). Walker (2013) recommended that family members should not be left alone during CPR. Rather, they should be supported and kept under supervision. None of these principles appears to have been followed during all of the relatives’ experiences, even with the relatives who were given the option to stay in the resuscitation room.

The relatives interviewed adopted various methods of support. Firstly, they valued the role of religion during their experience. All of them indicated the importance of religion as one of the main support systems during CPR. Several religious practices were used by relatives, such as reading from the Holy Quran and praying for their patient. Secondly, several relatives admitted trying to maintain hope as a method of self-supporting. They explained that feelings of hope gave them extra power to face their experiences.

…Absolutely, one of the main things that facilitate Muslims’ experiences is that believing and faith of Allah. Islam gives us explanations for everything about life and death… (Abu Yassin)

…There is always hope and miracles happen… (Abu Hammad)

The most important source of support for the participants was other family members. It was explained that patients’ close family rallied together and tried to encourage each other. This may be different in other cultures. All family members talked about the continuous presence of a large number of visitors for their loved one. The presence of close relatives, extended relatives, friends, and neighbours was viewed as source of social, psychological and financial support. The presence of all of these people provided several benefits for close relatives. These people tried to help close relatives by talking to them and reminding them of Islamic precepts. Other relatives provided financial support as well.

…People started to come within five minutes. During 20 minutes or let us say when he transferred into the operation, there were around 15 persons…they support us…the presence of people around us helped and supported us and decreased our pain and crisis… (Abu Isa)

It is often stated that support received from other family members is important. However, there is a need to focus more on the roles of hospitals and health professionals in supporting relatives of patients during CPR. These roles included giving information about the patient’s condition, answering relatives’ questions, being honest, and providing a good level of care for their patients. This contributes to improving the trust of family members in health professionals and hospitals generally. Om Adnan, for example, talked about the role of healthcare professionals in dealing with family members.

…There were some co-operative health professionals…I was asking these for help many times. I asked them to help me in controlling my mother’s psychological status … they were co-operative with me and this helped me and had a positive effect on me… (Om Adnan)

Several ways of supporting family members were suggested in the literature. The implementation of a bereavement program for family members was thought to improve the grieving process and increase satisfaction with the care being provided (Gutierrez, 2012). Staff should stay with family members, assess their coping abilities, explain all procedures to them, and remain with them after finishing the resuscitation (Walker, 2013). Verhaeghe et al. (2005) stated that family members of critically ill patients should be supported financially and emotionally. Other researchers focused on the right of family members to express their satisfaction with the level of support they received from the health professionals as a way to improve healthcare organisations and services (M. Weslien, Nilstun, Lundqvist, & Fridlund, 2005).

In summary, though many studies have been done about FWR, this study is the only one that considers families’ needs during CPR in an Arabic Muslim community. It was found that family members want to be informed about the real situation of their patient and prognosis possibilities. In this study, family members confirm their preference to stay beside their loved one during CPR. Uniquely, religion was viewed as an essential requirement for many relatives. Family members thought that they support patients religiously by being allowed to stay in the resuscitation room. Consistent with previous studies, this study found that family members receive support from other relatives. Family members need to be reassured by healthcare professionals and they need their questions to be answered.

## 4. Discussion

Family members’ attitudes toward FWR were examined qualitatively in three studies (Wagner, 2004; Marita Weslien et al., 2006; Woning, 1999). However, only one of these studies Wagner (2004) was conducted in the critical care environment. None of these studies specifically discussed families’ needs at this difficult time. This is the first comprehensive study conducted in an Arabic Muslim country and that utilized a qualitative design to examine family members’ needs during CPR and FWR outside of the West. This sheds light on the possibility of implementing FWR practice out of the Western countries. More importantly, it highlights the importance of considering cultural and religious differences when implementing FWR in a new arena.

Family members explained that religion is an essential part in the daily life of the Jordanian population. They indicated the importance of religion in formulating other life aspects such as the interaction between people, the relationships between family members, the interaction with illnesses and crisis, and the end-of-life issues. All family members identified religious reasons for their desires to stay beside their loved ones during CPR, such as praying for God to help and support their loved ones. These activities were thought to support patients and their relatives. No precedent in the literature examined the influence of religion on families’ needs during CPR. Despite this, the findings of this study reveal that people’s religion and beliefs should be considered when we deal with CPR and end-of-life care. Consistently, Andrews (2008) explained that “religious beliefs may influence a client’s explanations of the cause(s) of illness, perception of its severity, and choice of healer(s)” (p. 356). Andrews explained that religion may become more effective in case of serious illness, as it may become source of support for patients and family members, and it may influence the course of action believed to be appropriate. This view further explained by Leininger and McFarland (2002) and Halligan (2006) who identified the role of religion in shaping the worldviews of Arab Muslim people. Being conscious of the effect of religion on Arab Muslims would make patients and their relatives cooperate with and appreciate health professionals’ efforts. Therefore, health professionals need to have knowledge about the worldview of Islam as a cultural influence on the daily life of the people.

Muslims believe that death will not happen unless by God’s permission. There is an important Islamic rule stated in the Holy Qur’an, that “whoever killed a person will be as the one who killed all people, and whoever saved a person will be as the one who saved all people” (Al-Jaza’iry, 2001). In Islam it is not permitted to end the life of anybody and death will be acceptable after making every effort to save a patient’s life. This makes concepts like “do not resuscitate” problematic for most Muslims. These principles may explain Jordanian families’ desire to be present during their loved one’s CPR. Family members want to see that their relative had received the best treatment before dying. They also want to be sure that their relative’s dignity has been preserved, especially if the patient is female. Furthermore, they want to support their relative by praying and reading from the Holy Qur’an.

The family members in this study respected the cultural and social issues and they thought that these issues should be respected by healthcare professionals. Family members stated that it is a duty to visit critically ill patients and they thought that family members must be always around the patients. They thought that ‘big family size’ and ‘strong bond between family members’ are sources of support and a comfort for patients and the very closest relatives. However, some of them indicated the difficulties of controlling the situation with presence of large number of visitors around the patient. However, they suggested finding alternative solutions to deal with these issues rather than refusing them at all.

Trust is critical to patients’ willingness to seek care, reveal sensitive information, submit to treatment, and follow physicians’ recommendations (Hall, Dugan, Zheng, & Mishra, 2001). Trust is also important to families as well to patients. This may explain the views of some family members that they would attend their relative’s CPR if they did not trust the CPR team or the institution. Many of these family members said that they would assess the situation first, and then they would decide whether or not to attend the CPR.

It was clear that there was less support in the public and the university hospitals than the private ones. Family members receive better support in the private hospitals. This may explain the fact that Jordanian people prefer obtaining healthcare services from the best private sector hospitals. However, cost is a barrier for many Jordanians to do this. This is in agreement with the findings of Mrayan (2005), as she indicated that private hospitals have more resources than in public hospitals. This may create dissatisfaction and anger among critically ill patients and family members who are treated in public hospitals. They might think that better care is available in private hospitals. Despite all these factors, all the study participants expressed their needs for information, reassurance and support. However, it was clear that meeting these needs was easier in the private hospitals than in the public and university hospitals. Therefore, we recommend doing further research on this issue in Jordan. This includes comparing the quality of healthcare services in health sectors.

Despite the differences in education between the participants, they expressed similar needs during CPR of their loved ones. Family members stated that they could understand the procedures, especially if they were explained well. Some of the family members were angry with the way that healthcare professionals dealt with them during CPR. They thought that good explanations about the patient’s condition would improve the trust between family members and healthcare professionals. Generally, the more educated people were more confident about their right to stay beside their loved ones during CPR.

## 5. Conclusion

Uniquely, this study includes groups of family members after both successful and failed CPR. Some of the family members interviewed were given the option to witness the CPR while other did not have this choice. The findings of this study reveal that all family members found death and CPR very stressful. However, all of them explained that being with their loved one during CPR was better than staying outside the resuscitation room. Interestingly, the family members who were given this option said they would insist on staying if they faced a similar situation in the future.

The findings in this study show that most of the family members wanted to stay beside their loved ones during CPR. Many of them wanted this option for religious purposes. This study indicated that the most important need for Jordanian families was to be reassured and informed about the condition of their loved ones. Keeping families up to date about the prognosis of their loved one would increase trust in health professionals and would improve families’ acceptance of the final outcomes of the treatment process. Indeed all of these findings seem important, not in the Jordanian context, but also when dealing with people with similar characteristics. Respecting families’ beliefs and cultures are important during CPR or at the time of death. More importantly, family members should be involved in the treatment process and reassured about the condition of their loved one.

The findings of this study recommend more support and care for family members. This could be achieved by the presence of special staff to deal with patient’s relatives during CPR and at time of death. Support staff should communicate with the CPR team to control and organise FWR. It was found that critically ill patients’ family members are generally neglected during the treatment process. It may not be obligatory that family members are given the opportunity to witness resuscitation efforts, but we strongly suggest that hospitals should take into account the circumstances of the relatives during CPR. In terms of methodology, there is a need for more experimental and qualitative research with patients, family members, health professionals and policy makers. Where possible, a larger sample size is recommended.

## References

[ref1] Al-Hassan M. A, Hweidi I. M (2004). The perceived needs of Jordanian families of hospitalized, critically ill patients. International Journal of Nursing Practice.

[ref2] Al-Jaza’iry A (2001). Minhaj Al-Muslim.

[ref3] Al-Mutair A. S, Plummer V, Copnell B (2012). Family presence during resuscitation: a descriptive study of nurses’ attitudes from two Saudi hospitals. Nursing in Critical Care.

[ref4] Ambert A, Adler A, Adler P, Detzner F (1995). Understanding and Evaluating Qualitative Research. Journal of Marriage and the Family.

[ref5] (2006). American Heart Association. CPR facts and statistics. www.americanheart.org/presenter.

[ref6] Andreae D (2010). The Family Affliction: Mental Health and the Elderly.

[ref7] Andrews M, Andrews M, Boyle J (2008). Religion, culture, and nursing. Transcultural concepts in nursing care.

[ref8] Axelsson Å.B, Fridlund B, Moons P, Mårtensson J, op Reimer W. S, Smith K, Norekvåli T. M, the Undertaking Nursing Interventions Throughout Europe (UNITE) study group (2010). European Cardiovascular Nurses’ Experiences of and Attitudes Towards Having Family Members Present in the Resuscitation Room. European Journal of Cardiovascular Nursing.

[ref9] Bailey J. J, Sabbagh M, Loiselle C. G, Boileau J, McVey L (2010). Supporting families in the ICU: A descriptive correlational study of informational support, anxiety, and satisfaction with care. Intensive and Critical Care Nursing.

[ref10] Baskett P. J. F, Lim A (2004). The varying ethical attitudes towards resuscitation in Europe. Resuscitation.

[ref11] Baskett P. J. F, Steen P. A, Bossaert L (2005). European Resuscitation Council Guidelines for Resuscitation 2005: Section 8. The ethics of resuscitation and end-of-life decisions. Resuscitation.

[ref12] Blom H, Gustavsson C, Sundler A. J (2013). Participation and support in intensive care as experienced by close relatives of patients—A phenomenological study. Intensive and Critical Care Nursing.

[ref13] Braun V, Clarke V (2006). Using thematic analysis in psychology. Qualitative Research in Psychology.

[ref14] Bryman A (2012). Social Reasearch Mehtods.

[ref15] Brysiewicz P, Uys L. R (2006). A Model for Dealing With Sudden Death. Advances in Nursing Science.

[ref16] Davidson J. E, Jones C, Bienvenu O. J (2012). Family response to critical illness: Postintensive care syndrome – family. Critical Care Medicine.

[ref17] Davis L (1994). Perceived needs of families of long-term critical care patients: a brief report. Heart and Lung.

[ref18] Doyle C, Post H, Burney R, Maino J, Keefe M, Rhee K (1987). Family participation during resuscitation: an option. Annals of Emergency Medicine.

[ref19] Farrell M (1989). Dying and bereavement: The role of the critical care nurse. Intensive Care Nursing.

[ref20] Fulbrook P, Albarran J. W, Latour J. M (2005). A European survey of critical care nurses’ attitudes and experiences of having family members present during cardiopulmonary resuscitation. International Journal of Nursing Studies.

[ref21] Gamell A, Corniero P, Palazon P, Parra C, Trenchs V, Luaces C (2011). Parental presence during invasive procedures in a Spanish pediatric emergency department: incidence, perspectives, and related anxiety. European Journal of Emergency Medicine.

[ref22] Grice A, Picton P, Deakin C (2003). Study examining attitudes of staff, patients and relatives to witnessed resuscitation in adult intensive care units. British Journal of Anaesthesia.

[ref23] Gutierrez K. M (2012). Experiences and Needs of Families Regarding Prognostic Communication in an Intensive Care Unit: Supporting Families at the End of Life. Critical Care Nursing Quarterly.

[ref24] Hall M, Dugan E, Zheng B, Mishra A (2001). Trust in physicians and medical institutions: what is it, can it be measured, and does it matter?. The Milbank Quarterly.

[ref25] Halligan P (2006). Caring for patients of Islamic denomination: critical care nurses’ experiences in Saudi Arabia. Journal of Clinical Nursing.

[ref26] Halm M (2005). Family presence during resuscitation: a critical review of the literature. American Journal of Critical Care.

[ref27] Hanson B (2008). Wither Qualitative/Quantitative? Grounds for methodological convergence. Quality & Quantity.

[ref28] Hanson C, Strawser D (1992). Family presence during cardiopulmonary resuscitation: Foote Hospital emergency department's nine-year perspective. Journal of emergency nursing: JEN : official publication of the Emergency Department Nurses Association.

[ref29] Hinkle J. L, Fitzpatrick E (2011). Needs of American relatives of intensive care patients: Perceptions of relatives, physicians and nurses. Intensive and Critical Care Nursing.

[ref30] Holden J, Harrison L, Johnson M (2002). Families, nurses and intensive care patients: a review of the literature. Journal of Clinical Nursing.

[ref31] Hupcey J (1999). Looking out for the patient and ourselves: the process of family integration into the ICU. Journal of Clinical Nursing.

[ref32] Jamerson P, Scheibmeir M, Bott M, Crighton F, Hinton R, Kuckelman Cobb A (1996). The experiences of families with a relative in the intensive care unit. Heart and Lung.

[ref33] Khoury S, Massad D, Fardous T (1999). Mortality and causes of death in Jordan 1995-96: assessment by verbal autopsy. Bulletin of the World Health Organization.

[ref34] Kopelman A (2006). Understanding, avoiding, and resolving end-of-life conflicts in the NICU. The Mount Sinai Journal of Medicine.

[ref35] Lazarus R. S, Folkman S (1984). Stress, appraisal, and coping / Richard S. Lazarus, Susan Folkman.

[ref36] Lee L, Lau Y (2003). Immediate needs of adult family members of adult intensive care patients in Hong Kong. Journal of Clinical Nursing.

[ref37] Leininger M, McFarland M (2002). Transcultural nursing: concepts, theories, research and practice.

[ref38] Madden E, Condon C (2007). Emergency Nurses’ Current Practices and Understanding of Family Presence During CPR. Journal of Emergency Nursing.

[ref39] Mangurten J, Scott S. H, Guzzetta C. E, Clark A. P, Vinson L, Sperry J, Voelmeck W (2006). Effect of family presence during resuscitation and invasive procedures in a pediatric emergency department. Journal of Emergency Nursing.

[ref40] Marrone L, Fogg C (2003). Should the family be present during resuscitation?. Nursing.

[ref41] Mason J (2002). Qualitative research.

[ref42] Maxwell J (2012). Qualitative research design: An interactive approach.

[ref43] McKiernan M, McCarthy G (2010). Family members’ lived experience in the intensive care unit: A phemenological study. Intensive and Critical Care Nursing.

[ref44] Meyers T, Eichhorn D, Guzetta C, Clark A, klein J, Taliaferro E, Klein J. D (2000). Family presence during invasive procedures and resuscitation: the experience of family members, nurses, and physicians. American Journal of Nursing.

[ref45] Molter N (1979). Needs of relatives of critically ill patients: a descriptive study. Heart and Lung.

[ref46] Moreland P, Manor B (2005). Family presence during invasive procedures and resuscitation in the emergency department: a review of the literature. Journal of Emergency Nursing.

[ref47] Mrayyan M. T (2005). Nurse job satisfaction and retention: comparing public to private hospitals in Jordan. Journal of Nursing Management.

[ref48] Ong M, Chan Y, Srither D, Lim Y (2004). Asian medical staff attitudes towards witnessed resuscitation. Resuscitation.

[ref49] Polit D, Beck C (2010). Essentials of Nursing Research: Appraising Evidence for Nursing Practice.

[ref50] Redley B, Botti M, Duke M (2004). Family member presence during resuscitation in the emergency department: an Australian perspective. Emergency Medicine Australasia.

[ref51] Strong P (1992). The case for qualitative research. International Journal of Pharmacy Practice.

[ref52] (2006). USAID in Jordan. About Jordan. http://www.usaidjordan.org/jordan.cfm.

[ref53] Verhaeghe S, Defloor T, Zuuren F, Duijnstee M, Grypdonck M (2005). The needs and experiences of family members of adult patients in an intensive care unit: a review of the literature. Journal of Clinical Nursing.

[ref54] Wagner J. M (2004). Lived Experience of Critically Ill Patients’ Family Members During Cardiopulmonary Resuscitation. Am J Crit Care.

[ref55] Walker W (2008). Accident and emergency staff opinion on the effects of family presence during adult resuscitation: critical literature review. Journal of Advanced Nursing.

[ref56] Walker W. M (2013). Emergency care staff experiences of lay presence during adult cardiopulmonary resuscitation: a phenomenological study. Emergency Medicine Journal.

[ref57] Walters A (1995). A hermeneutic study of the experiences of relatives of critically ill patients. Journal of Advanced Nursing.

[ref58] Wanger J. M (2004). lived experienc of critically ill patients’ family members during cardiopulmonary resuscitation. American Journal of Critical Care.

[ref59] Weissman D. E (2004). Decision Making at a Time of Crisis Near the End of Life. JAMA.

[ref60] Weslien M, Nilstun T, Lundqvist A, Fridlund B (2005). When the unreal becomes real: family members’ experiences of cardiac arrest. Nursing in Critical Care.

[ref61] Weslien M, Nilstun T, Lundqvist A, Fridlund B (2006). Narratives about resuscitation--Family members differ about presence. European Journal of Cardiovascular Nursing.

[ref62] Woning M. V. d (1999). Relatives in the resuscitation area: a phenomenological study. Nursing in Critical Care.

